# A Machine Learning Approach for the Prediction of Testicular Sperm Extraction in Nonobstructive Azoospermia: Algorithm Development and Validation Study

**DOI:** 10.2196/44047

**Published:** 2023-06-21

**Authors:** Guillaume Bachelot, Ferdinand Dhombres, Nathalie Sermondade, Rahaf Haj Hamid, Isabelle Berthaut, Valentine Frydman, Marie Prades, Kamila Kolanska, Lise Selleret, Emmanuelle Mathieu-D’Argent, Diane Rivet-Danon, Rachel Levy, Antonin Lamazière, Charlotte Dupont

**Affiliations:** 1 Saint Antoine Research Center L'Institut national de la santé et de la recherche médicale UMR 938 Sorbonne Université Paris France; 2 Service de Biologie de La Reproduction Hôpital Tenon Assistance Publique-Hôpitaux de Paris, Sorbonne Université Paris France; 3 Laboratory in Medical Informatics and Knowledge Engineering in e-Health L'Institut national de la santé et de la recherche médicale Sorbonne University Paris France; 4 Service d’Urologie Hôpital Tenon Assistance Publique-Hôpitaux de Paris, Sorbonne Université Paris France; 5 Service de Gynécologie Obstétrique et Médecine de la Reproduction Hôpital Tenon Assistance Publique-Hôpitaux de Paris, Sorbonne Université Paris France; 6 Département de Métabolomique Clinique Hôpital Saint Antoine Assistance Publique-Hôpitaux de Paris, Sorbonne Université Paris France

**Keywords:** machine learning, azoospermia, prediction model, biomedical informatics, model, predict, sperm, men's health, infertility, infertile

## Abstract

**Background:**

Testicular sperm extraction (TESE) is an essential therapeutic tool for the management of male infertility. However, it is an invasive procedure with a success rate up to 50%. To date, no model based on clinical and laboratory parameters is sufficiently powerful to accurately predict the success of sperm retrieval in TESE.

**Objective:**

The aim of this study is to compare a wide range of predictive models under similar conditions for TESE outcomes in patients with nonobstructive azoospermia (NOA) to identify the correct mathematical approach to apply, most appropriate study size, and relevance of the input biomarkers.

**Methods:**

We analyzed 201 patients who underwent TESE at Tenon Hospital (Assistance Publique-Hôpitaux de Paris, Sorbonne University, Paris), distributed in a retrospective training cohort of 175 patients (January 2012 to April 2021) and a prospective testing cohort (May 2021 to December 2021) of 26 patients. Preoperative data (according to the French standard exploration of male infertility, 16 variables) including urogenital history, hormonal data, genetic data, and TESE outcomes (representing the target variable) were collected. A TESE was considered positive if we obtained sufficient spermatozoa for intracytoplasmic sperm injection. After preprocessing the raw data, 8 machine learning (ML) models were trained and optimized on the retrospective training cohort data set: The hyperparameter tuning was performed by random search. Finally, the prospective testing cohort data set was used for the model evaluation. The metrics used to evaluate and compare the models were the following: sensitivity, specificity, area under the receiver operating characteristic curve (AUC-ROC), and accuracy. The importance of each variable in the model was assessed using the permutation feature importance technique, and the optimal number of patients to include in the study was assessed using the learning curve.

**Results:**

The ensemble models, based on decision trees, showed the best performance, especially the random forest model, which yielded the following results: AUC=0.90, sensitivity=100%, and specificity=69.2%. Furthermore, a study size of 120 patients seemed sufficient to properly exploit the preoperative data in the modeling process, since increasing the number of patients beyond 120 during model training did not bring any performance improvement. Furthermore, inhibin B and a history of varicoceles exhibited the highest predictive capacity.

**Conclusions:**

An ML algorithm based on an appropriate approach can predict successful sperm retrieval in men with NOA undergoing TESE, with promising performance. However, although this study is consistent with the first step of this process, a subsequent formal prospective multicentric validation study should be undertaken before any clinical applications. As future work, we consider the use of recent and clinically relevant data sets (including seminal plasma biomarkers, especially noncoding RNAs, as markers of residual spermatogenesis in NOA patients) to improve our results even more.

## Introduction

In the context of azoospermia, testicular sperm extraction (TESE) can be proposed to obtain mature germ cells (ie, spermatozoa) for in vitro fertilization with intracytoplasmic sperm injection (ICSI) [1-3]. Several surgical techniques are available for this, including conventional surgical TESE (cTESE) and microsurgical TESE (microTESE), the latter of which requires the use of an operating microscope to visualize the seminiferous tubules that are most likely to contain complete spermatogenesis [4]. However, both of these are invasive procedures and are, thus far, not exempt from complications such as hematoma, infection, vascular damage, and testosterone deficiency [4]. Thus, TESE must be proposed after a couple’s complete infertility checkup and information session as well as following a multidisciplinary discussion. Finally, spermatozoa can be retrieved from testicular tissue in only about 50% of cases [5,6].

Some teams have aimed to identify the most predictive factors of a positive TESE, first using univariate models, followed by multivariate models and, finally, artificial intelligence, including machine learning (ML) models [7]. We conducted a scoping review on the prediction of TESE success, extended to relevant citations on PubMed (MeSH terms: TESE; prediction; non-obstructive azoospermia; machine learning; sperm). A combination of search terms and Boolean operators (such as OR, AND) were used as appropriate to broaden the search and retrieve all relevant papers.

Among the clinical and hormonal variables in a preoperative assessment, age, BMI, total testosterone, and prolactin levels seem insufficiently predictive of the presence of spermatozoa in testicular tissue [8-13], while small testicular volume [14-17]; high follicle-stimulating hormone (FSH) [18]; and low inhibin B, reflecting impaired testicular function, are generally accompanied by a lower probability of successful TESE [19-22]. In addition, abnormal karyotype and microdeletion in the azoospermia factor (AZF) region are found in 6% to 18% of patients with azoospermia [23-26]. Except for complete AZFa and AZFb mutations, which are systematically associated with an absence of complete spermatogenesis, genetic results are insufficient predictors of TESE outcomes [26]. Finally, a history of cryptorchidism and smoking status have shown inconsistent results [27-29]. Consequently, separately, none of these predictors has so far shown satisfactory, sufficient, and reproducible predictive performance to guide practitioners regarding the probability of TESE success and the evaluation of the benefit-risk balance [7,14,30-33]. In addition, this surgery involves a substantial cost for some patients.

ML techniques allow for predictions based on the integration of a large amount of data due to an increase in computing power. We previously reported the relevance of mathematical modeling and ML approaches [34,35]. Concerning TESE, different models were developed with conventional clinical and biological data from the preoperative checkup. Mostly, the models developed were logistic regression (LR) or artificial neural network (ANN) models. For instance, Tsujimura et al [36] developed an LR using 100 patients and an area under the curve (AUC) of 0.83. Notwithstanding the use of a larger cohort of more than 1000 patients, the LR model from Cissen et al [37] achieved an AUC of only 0.65. Furthermore, the ANN model that Ramasamy et al [38] used yielded even poorer performance, with an AUC of 0.59. Finally, Zeadna et al [39] developed a more complex ensemble model based on decision trees (XGBoost [XGB]), which exhibited a sensitivity of over 90% but a very low specificity (51%).

Nevertheless, these studies applied different approaches and used various sample sizes, making it difficult to compare them and establish the correct mathematical methodology.

As it is challenging to determine which mathematical approach and, thus, which type of ML model will be the most appropriate and effective, an extensive search of the most common models is essential to avoid model selection bias. Similarly, the number of patients needed to obtain a successful model is mostly empirical and depends on the problem and type of data made available.

Therefore, the aim of this study was to develop, evaluate, and compare a wide range of predictive models under similar conditions for TESE outcomes in patients with nonobstructive azoospermia (NOA) to identify the correct mathematical approach to apply thereto, as well as the most appropriate study size. To our knowledge, our study is the first intermethodology comparative study in this field, as well as the first to assess the requisite sample size.

## Methods

### Description of the Patients

Data from 201 patients who underwent TESE (cTESE or microTESE) between January 2012 and December 2021 at Tenon Hospital (Assistance Publique-Hôpitaux de Paris, Sorbonne University, Paris) were collected. All included patients presented with NOA, which is defined as the absence of spermatozoa in the semen in at least two collections at least three months apart (2010 World Health Organization criteria for semen analysis) [40]. Patients with NOA following radiotherapy or with hypogonadotropic hypogonadism were excluded from the study.

### Surgical Procedure

The surgical procedure was systematically performed under general anesthesia. After the scrototomy and opening of the tunica vaginalis, the albuginea was incised. The testicular pulp was then expressed and dissected (cTESE: 136/201, 67.7%). In some cases, the dissection of testicular tissue was done under a surgical microscope to better locate the seminiferous tubules that most likely contain sperm (microTESE: 65/201, 32.3%). Fragments could be taken bilaterally (from both testicles) or unilaterally at the upper, middle, and lower pole of each testicle, sparing the rete testis area. One sample was systematically collected to send it for anatomopathology. Once collected, the testicular fragments were transported in a culture medium (Ferticult Hepes) to the laboratory. The testicular fragments were then prepared using mechanical methods and observed under optical microscopy for the spermatozoon search [40].

### Study Overview

**Figure 1:**
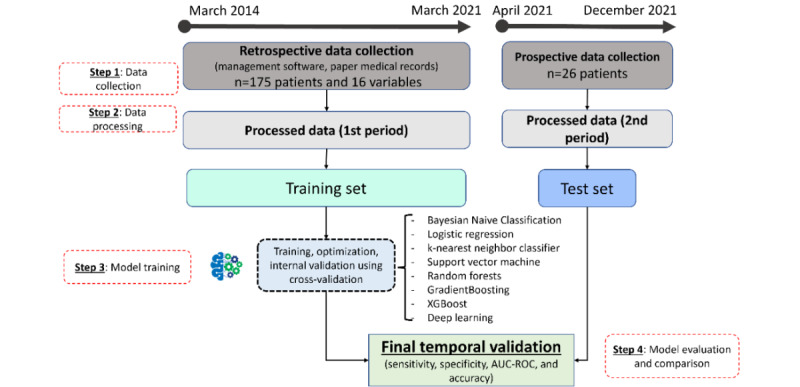
Summary workflow from data collection to final model evaluation. AUC-ROC: area under the receiver operating characteristic curve.

In the first step, preoperative data including urogenital history, hormonal data, genetic data, and TESE outcomes (representing the target variable) were collected from patients’ medical records, including management software and paper records (patients in the retrospective training cohort: 175/201, 87.1%; patients in the prospective testing cohort: 26/201, 12.9%). This data collection was driven by the potentially relevant predictors identified from the literature and expert recommendations regarding evaluation of male infertility [6,41,42]. In the second step, raw data were preprocessed (eg, imputation of missing values, encoding [eg, turning qualitative variable into numbers], and scaling [eg, transforming quantitative variable when they were on a different scale]) to transform them into a format suitable for the ML models. Once these data were preprocessed, 8 ML and deep learning (DL) models were trained and selected using the retrospective training cohort data in the third step. Finally, in the 4th and last step, data from the prospective training cohort were used to evaluate the models (temporal validation) and allow for comparison using the prospective test set from patients the models had never seen. The results from the test set give a good indication of how the model should perform in the real world (Figure 1).

### Data Set

#### Description of the Variables

The input data corresponded with the French standard exploration of male infertility and the TESE preoperative assessment [6]. Among the 16 included variables, 7 were quantitative, and 9 were qualitative or categorical. The variables were age, BMI, tobacco consumption, hormonal assessment (FSH, luteinizing hormone [LH], testosterone, inhibin B, and prolactin), genetic exploration (karyotype and search for Y-chromosome microdeletion), and urogenital history (cryptorchidism, infection, trauma, gonadotoxic therapy, urogenital surgery, and varicoceles), for a total of 16 variables.

#### Description of the Target

The outcome was the presence (y=1) or absence (y=0) of spermatozoa after examination of the surgical specimens. A TESE was considered positive when we obtained enough spermatozoa for the ICSI procedure.

### Statistical Analysis

Prior to the modeling process, exploratory analysis of the data was conducted to quantify missing data and analyze the variables’ distributions. Correlations and mean comparisons were also performed. Regarding the statistics, the qualitative variables are reported as percentages, and the quantitative variables are reported as medians (IQRs). We used the Mann-Whitney test for quantitative variables (among the quantitative variables, none was normally distributed [Shapiro-Wilk]) and χ^2^ for qualitative variables. For correlations, the Pearson coefficient was used. A *P* value of <.05 was considered significant.

### Preprocessing and Modeling

Our main objective was to train a classification model to classify a new patient as presenting with either an “absence of sperm” or “presence of sperm” during TESE based on the patient’s variables. After processing the raw data (eg, handling missing data, management of categorical or qualitative variables, standardization), many different models (n=8) were trained, optimized, and evaluated. ML models (Bayesian naive classification, LR, k-nearest neighbor classifier, support vector machine [SVM], random forest [RF], gradient-boosted tree [GBT], and XGB) and DL models using several neural network architectures were also tested. The optimization and fine-tuning of the hyperparameters were performed using a cross-validation random search technique on each split of the training set: Each hyperparameter was sampled from a distribution of possible parameter values. The highest performing hyperparameter combination was used for each model [43].

### The Validation and Evaluation Procedure

The data set (patient cohort) was distributed into a training set for training, during which hyperparameters were set and models were selected (internal validation, repeated 5-fold cross-validation [10 iterations]), and a separate test set, during which a final evaluation of patients that the models had never seen (external validation) was conducted. The external validation consisted of testing the models’ ability to classify or correctly predict the patients of the test cohort. Both sets came from the same institution, but the patients’ data were collected at separate periods. In a monocentric study, splitting by time and developing a model using data from one period and evaluating its performance using the data from the other period (temporal validation) is a stronger approach, as indicated by the “Transparent reporting of a multivariable prediction model for individual prognosis or diagnosis” (TRIPOD) Statement [44].

The following several classification metrics were used to evaluate the models and compare them objectively: sensitivity, specificity, area under the receiver operating characteristic curve (AUC-ROC), and accuracy. Moreover, as a binary classification task, the thresholds for both ML and deep learning models were maintained at 50%. For example, a TESE was classified as positive when the probability of success was greater than 50% and negative when the probability was less than 50%. Finally, the following 2 additional factors were considered when evaluating the models: importance of each variable in the model and optimal number of patients to include in the study. The importance of each variable in the models was determined using the permutation technique: Permutation feature importance (PFE) consists of comparing the performances of the model with and without the variable under evaluation [45]. PFE generates an ordered list of variables along with their importance values. Interpreting the output of this algorithm is straightforward‎: Variables ranked higher have more impact on the model predictions. Identifying relevant variables is also useful for the model’s explainability. For the optimal number of patients, we used the learning curve, which is a graphical representation of the relationship between the model’s performance (measured on the vertical axis) and the number of patients used for training (measured on the horizontal axis). A learning curve allows for visually evaluating the evolution of the model’s performance as new patients are included. The shape of this learning curve (plateau or rising slope) provides information on the number of patients needed to obtain good performance and discern whether it is necessary to include new patients.

### Computer Tools

The whole project was realized exclusively with the Python 3.8 programming language and several libraries: NumPy 1.20.3 and Pandas 1.2.4 for data table management and matrix calculation, SciPy 1.7.0 and Pingouin 0.3.12 for statistics, and Sklearn 0.24.2 and TensorFlow 2.5.0 for modeling [46-50]. The code to build and train the model is openly available on github [51].

### Ethical Considerations

The local ethics committee “Comité d’Éthique de la Recherche, Sorbonne Université” (CER@SU) approved this study under protocol number CER-2021-041, and this study had no external source of funding.

## Results

### Exploratory Data Analysis

The clinical characteristics of the patients included in the study are shown in Table 1. All the variables from the preoperative assessment were included, with missing data in the training cohort ranging from 5.7% (10/175) to 57.7% (101/175) for varicocele and prolactin, respectively. No missing data were reported in the test cohort. Within the training set (n=175), enough spermatozoa for the ICSI procedure were found in the testicular tissue of 104 (59.4%) patients (positive TESE), while the TESE was negative in the 71 (40.6%) remaining patients. Within the test set, the distribution between the classes was equal. The following 3 variables were significantly different between the 2 groups: FSH, LH, and inhibin B levels (Table 1). In addition, in the training set, several quantitative variables were significantly correlated: FSH and inhibin B (*r*=−0.637; *P*<.001), LH and inhibin B (*r*=−0.454; *P*<.001), LH and testosterone (*r*=−0.194; *P*=.03), age and LH (*r*=−0.186; *P*=.04), FSH and total testosterone (*r*=−0.176; *P*=.04), age and inhibin B (*r*=0.222; *P*=.04), and FSH and LH (*r*=0.852; *P*<.001).

**Table 1:** Baseline characteristics of patients in the training and test sets.

Variable					Presence of spermatozoa (n=104)			Absence of spermatozoa (n=71)		*P* value
**Training set (n=175)**										
	**Clinical**									
			Age^a^ (years), median (IQR)	38.11 (9.56)			36.89 (5.40)		.16	
			BMI^b^ (kg/m^2^), median (IQR)	25.41 (4.99)			25.11 (3.65)		.87	
			Smoking^c^ (yes), n (%)	27^d^ (28.7)			19^e^ (30.2)		.99	
	**Hormonal**									
			FSH^f,g^ (UI/L), median (IQR)	9.90 (14.10)			23.50 (23.03)		<.001	
			LH^h,i^ (UI/L), median (IQR)	6.37 (5.49)			11.90 (12.80)		<.001	
			Testosterone^i^ (ng/mL), median (IQR)	4.50 (2.76)			4.00 (3.05)		.28	
			Inhibin B^j^ (pg/mL), median (IQR)	60.00 (94.00)			14.00 (28.25)		<.001	
			Prolactin^k^ (ng/mL), median (IQR)	12.00 (5.58)			11.85 (8.08)		.94	
	**Genetics**									
			Normal karyotype^l^, n (%)	9^m^ (10.3)			6^n^ (9.4)		>.99	
			Y microdeletion^o^, n (%)	2^p^ (2.1)			3^n^ (4.7)		.61	
	**Medical history**									
			Cryptorchidism^q^, n (%)	20^r^ (20.4)			11^s^ (16.4)		.66	
			Infection^t^, n (%)	11^u^ (11.3)			3^v^ (4.6)		.23	
			Trauma^w^, n (%)	2^u^ (2.1)			1^x^ (1.5)		>.99	
			Gonadotoxic therapy^w^, n (%)	8^r^ (8.2)			7^v^ (10.8)		.77	
			Urogenital surgery^t^, n (%)	11^u^ (11.3)			6^v^ (9.2)		.87	
			Varicocele^q^, n (%)	14^y^ (14.1)			15^x^ (22.7)		.23	
**Test set (n=26)**										
	**Clinical**									
		Age (years), median (IQR)		40.83 (6.50)		36.62 (4.75)			.09	
		BMI (kg/m^2^), median (IQR)		25.95 (3.72)		29.12 (6.42)			.25	
		Smoking (yes), n (%)		3^z^ (23.1)		5^z^ (38.5)			>.99	
	**Hormonal**									
		FSH (UI/L), median (IQR)		8.59 (5.58)		25.78 (9.76)			<.001	
		LH (UI/L), median (IQR)		6.67 (3.69)		10.88 (4.42)			.02	
		Testosterone (ng/mL), median (IQR)		4.30 (3.37)		4.45 (2.85)			.45	
		Inhibin B (pg/mL), median (IQR)		109.00 (92.10)		20.00 (18.00)			<.001	
		Prolactin (ng/mL), median (IQR)		10.00 (3.38)		8.00 (3.60)			.98	
	**Genetics**									
		Normal karyotype, n (%)		1^z^ (7.7)		1^z^ (7.7)			.99	
		Y microdeletion, n (%)		1^z^ (7.7)		0^z^ (0)			.99	
	**Medical history**									
		Cryptorchidism, n (%)		3^z^ (23.1)		1^z^ (7.7)			.59	
		Infection, n (%)		0^z^ (0)		1^z^ (7.7)			.98	
		Trauma, n (%)		0^z^ (0)		0^z^ (0)			.99	
		Gonadotoxic therapy, n (%)		0^z^ (0)		0^z^ (0)			.99	
		Urogenital surgery, n (%)		0^z^ (0)		0^z^ (0)			.99	
		Varicocele, n (%)		2^z^ (15.4)		1^z^ (7.7)			.97	

^a^25 (14.3%) missing values.

^b^43 (25.7%) missing values.

^c^18 (10.3%) missing values.

^d^N=94.

^e^N=63.

^f^FSH: follicle-stimulating hormone.

^g^17 (9.7%) missing values.

^h^LH: luteinizing hormone.

^i^37 (21.1%) missing values.

^j^74 (42.3%) missing values.

^k^101 (57.7%) missing values.

^l^14 (8%) missing values.

^m^N=87.

^n^N=64.

^o^15 (8.6%) missing values.

^p^N=96.

^q^10 (5.7%) missing values.

^r^N=98.

^s^N=67.

^t^13 (7.4%) missing values.

^u^N=97.

^v^N=65.

^w^12 (6.9%) missing values.

^x^N=66.

^y^N=99.

^z^N=13.

### The Valuation Procedure and Cohort Splitting

Both cohorts (training and test sets) were from the same institution, and patients were managed in a similar manner. A retrospective cohort of 175 patients was first used for model building. Following this first step of the study, a prospective collection of data of patients undergoing TESE in the institution was conducted. This second temporal test cohort, consisting of prospectively collected data from May 2021 to December 2021, served to independently evaluate the performance of the models. Moreover, the prospective data collection allowed the inclusion of only “complete” patients’ TESE preoperative assessments, resulting in no missing values for this temporal test cohort. The distribution between classes of 1/0 (positive/negative) was 104/71 in the training set and 13/13 in the test set. Further, the characteristics (variables) of the patients were not significantly different between the 2 cohorts (training and test).

### Performance of the Tested Models

Table 2 reports the internal and external validation results for the different models: The ensemble models based on decision trees (RF and GBT) showed the best performance. Additionally, the DL models were less efficient than the more classical ML models, especially in the test cohort. The highest performing hyperparameter combination for each model is reported in Table S1 in Multimedia Appendix 1.

**Table 2:** Internal and external performances of the 8 different trained models.

Model	Internal validation (cross validation), mean (SD)					External temporal validation			
	AUC-ROC^a^	Accuracy (%)	Sensitivity (%)	Specificity (%)	AUC-ROC		Accuracy (%)	Sensitivity (%)	Specificity (%)
LR^b^	0.670 (0.097)	66.4 (7.1)	76.3 (10.1)	53.7 (9)	0.85		69.2	76.9	61.5
BNC^c^	0.624 (0.092)	62.1 (7.5)	66.3 (11.5)	55.9 (11.3)	0.83		69.2	69.2	69.2
RF^d^	0.780 (0.084)	74.7 (7.3)	85.2 (7.1)	61 (11.5)	0.90		84.6	100.0	69.2
GBT^e^	0.765 (0.092)	73.5 (6.9)	82.2 (7.8)	62.4 (10.8)	0.82		76.9	92.3	61.5
XGB^f^	0.760 (0.087)	72.2 (7.1)	80.2 (7.8)	62.1 (9.9)	0.82		80.8	92.3	69.2
SVM^g^	0.723 (0.094)	67.6 (8.3)	81.8 (9.8)	49.1 (13.6)	0.72		61.5	84.6	38.5
KNN^h^	0.669 (0.089)	63.8 (6.1)	76.2 (9)	48.3 (11.1)	0.76		69.2	84.6	53.8
ANN^i^ (64x32)	0.690 (0.085)	66.7 (7)	77 (8.6)	55.4 (10.9)	0.65		65.0	54.0	77.0

^a^AUC-ROC: area under the receiver operating characteristic curve.

^b^LR: logistic regression.

^c^BNC: Bayesian naive classification.

^d^RF: random forest.

^e^GBT: gradient-boosted tree.

^f^XGB: XGBoost.

^g^SVM: support vector machine.

^h^KNN: k-nearest neighbor.

^i^ANN: artificial neural network.

### Top Model, Biomarker Ranking, and Sample Size Requirements

**Figure 2:**
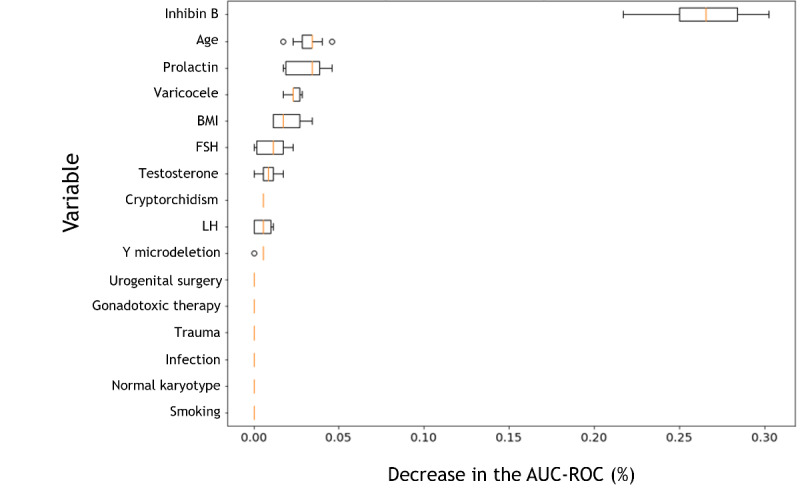
The importance of each variable in the random forest (RF) model using permutation feature importance (PFE), which generates an ordered list of variables along with their importance values: Variables with higher ranks have more impact on the model predictions. AUC-ROC: area under the receiver operating characteristic curve; FSH: follicle-stimulating hormone; LH: luteinizing hormone.

The most efficient model was the RF model. The RF model showed the best performance in the test cohort, with an AUC of 0.90, a sensitivity of 100%, and a specificity of 69.2%. Notably, the performances in the test cohort were better each time than during cross-validation in the training cohort. The RF model is an ensemble model (ie, it combines several models). For the RF model, the most discriminating variable (mean 26.6%, SD 2.5%) was serum inhibin B concentration (Figure 2). In addition, serum prolactin concentration (mean 3.1%, SD 1.1%), patient age (mean 3.2%, SD 0.8%), and the presence of a history of varicoceles (whatever the grade; mean, 2.3%, SD 0.4%) also seemed to be important in discriminating between the 2 groups.

Despite a lower reliability of the analysis, given the weaker performance of the 6 other models tested, the importance of the variables was also determined for 6 models (see Table S2 in Multimedia Appendix 2). These outputs were not available for neural networks (due to their black box appearance). Briefly, as shown in Table S2 in Multimedia Appendix 2, for the 2 decision tree models (XGB and GBT), which are similar to the RF model, the discriminant variables were comparable to those observed for the RF model (including serum inhibin and prolactin concentrations). In contrast, for linear models (such as SVM and LR), additional variables such as FSH concentration, smoking, and the presence of genetic abnormalities (Y microdeletion and abnormal karyotype) appeared to be also stratifying. However, the interpretation of the ranking results of these latter models should be interpreted with caution, insofar as such variable combinations did not produce efficient models.

**Figure 3:**
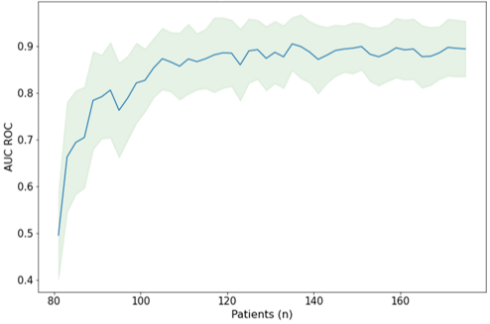
Learning curve for the random forest model, showing the graphical representation of the relationship between model performance (area under the receiver operating characteristic curve [AUC ROC]) and the number of patients used for training.

The performance of the RF model increased to a plateau at around 120 patients (Figure 3). Beyond this number of patients used to train the model, increasing the number of patients did not bring any performance gain. Indeed, beyond this threshold, the inclusion of additional patients did not allow for any increase in performance. These findings are similar for the other models evaluated: indeed, the learning curves also reached a plateau after 120 patients, showing the limitation of their performance despite the increase in the amount of data (see Multimedia Appendix 3). However, these results should be interpreted carefully, considering the poor relevance and performance of these models.

## Discussion

### Overview

We aimed to develop and evaluate predictive models of TESE outcomes in patients with NOA using ML methods and integrating retrospective data from the preoperative assessment. To our knowledge, this study was the first to compare several methodologies under the same conditions with the aim of determining the correct mathematical approach to apply and the most appropriate study size. TESE is an essential therapeutic tool for the management of male infertility and is often the “last hope” before gamete donation for these patients, but it is an invasive procedure. Consequently, it is essential to have a decision tool that could help the surgeon to decide whether this invasive procedure is the best option for the patient.

### Best Mathematical Approach to Apply and Best Sample Size to Use

Heterogeneity in the methodology and sample sizes in previous studies was the main obstacle to determining the correct mathematical approach to apply and the proper number of patients to include. In our study, the application of various approaches to the same cohort of patients allowed for an unbiased comparison. Regarding the different models investigated, the best results were provided by decision tree–based ensemble models, especially the RF model. Zeadna et al [39], who used the GBT and XGB models, which are close to the RF model, also found interesting results. Linear models such as LR or Bayesian models performed less well and produced results close to those of Tsujimura et al [36] and Cissen et al [37]. Despite more recent developments in artificial intelligence and more sophisticated technology, the neural network–based DL models yielded poor results that were below those of more traditional or classical methods. This could have been because data from the preoperative assessment used as input were inappropriate or because neural networks are classically used to process less structured, complex, and larger sized data such as sound signals or images (computer vision) or text (natural language processing). Overall, nonlinear methods such as decision trees and related methods (particularly the RF model) seem to be the most appropriate approach to predict TESE success. Beyond the raw performance of the models, the main strength of this study was its comparison of many models under identical conditions: similar patients and a similar sampling strategy. On the other hand, 120 patients seemed to be enough to properly exploit the preoperative data during the modeling process.

### The Relevance of Input Biomarkers

The most relevant variables in the previous models described in the literature were somewhat heterogeneous; for example, hormonal parameters (eg, LH, FSH) were powerful in the LR used by Cissen et al [37], while semen volume and ethnicity surprisingly appeared to be significant in the GBT used by Zeadna et al [39]. PFE measures the predictive value of a feature by evaluating how the prediction error increases when a variable is not available. PFE is a global explanation method that provides insights into an ML model’s behavior. It estimates and ranks feature importance based on the impact each variable has on the trained ML model’s predictions. In our study, and using the RF model, the most discriminating variable was the serum inhibin B level. Inhibin B is a hormone produced by Sertoli cells and is directly correlated with spermatogenesis. This hormone is a relevant variable in patients with and without spermatozoa [52]. Moreover, albeit with no significant difference between the groups, prolactin did indeed negatively impact sperm production [53], and therefore, its contribution to the discriminative power of the model seems meaningful. Unfortunately, due to missing data, we could not demonstrate the discriminative contribution of prolactin levels. Finally, the presence of a history of varicoceles, which is characterized by the dilation of a vein in the spermatic cord and is frequently found in infertile men, also seemed to be important in discriminating patients with positive TESEs from those with negative TESEs. Despite the good performance of the RF, the true classification power of conventional preoperative assessment can be challenged to explain the results of TESE. Moreover, the preoperative data were chosen for their accessibility and availability for incorporation into the patient’s therapeutic management. The selection of these data brings up other more general discussion points. For example, the available preoperative data may not have been sufficient to characterize the phenomenon that makes a TESE positive or not. Consequently, other variables or innovative biomarkers, such as genetic, proteomic, lifestyle, and environmental data, could be considered for further investigation. In the future, the integration of new biomarkers could allow for the construction of more efficient models [54]. These include serum 17-hydroxyprogesterone concentration, which appears to be an interesting marker of intratesticular testosterone [55]. More specifically, biomarkers in seminal plasma may be of interest. Thus, the integration of seminal inhibin B and seminal antiMüllerian hormone into an LR model could allow for the better prediction of the outcome of TESE [56]. Moreover, small noncoding RNA are increasingly being studied, and their variation in seminal plasma could encourage new diagnostic perspectives. For instance, Fang et al [57] observed different miRNA profiles in the seminal plasma of men with positive or negative TESEs. Likewise, Xie et al [58] were interested in the predictive characteristics of extracellular vesicle long noncoding RNA in seminal plasma. Finally, Ji et al [59] recently highlighted the value of circRNA for the same purpose.

### Limitations and Routine Usage of the Predictive Model

Two limitations of the study are worth mentioning: its monocentric design and the use of 2 surgical procedures, namely cTESE and microTESE. Despite the monocentric design, the work was carried out in 2 periods: first, model building with retrospective data from patients undergoing TESE until June 2021. This modeling process highlighted the challenge of collecting retrospective data and promoted the initiation of a prospective collection of patients managed from that date onwards. Following several months of data collection (until December 2021), a prospective testing cohort was therefore used to objectively evaluate the performance of the models. One perspective for the future should be to keep going in this way, including new patients over time and incorporating them into the model, in order to refine the performance of the models through time. The target could also have been a limitation, as its results were variable. Indeed, the target may depend on the surgeon, surgical technique used, site of the biopsy, and presence of focal spermatogenesis that may vary according to the tissue heterogeneity of the testis [60]. This heterogeneous distribution of seminiferous tubules, making the result dependent on the skill and experience of the surgeon, can eventually lead to errors in patient labeling, which can be detrimental to building ML models. Furthermore, the development of the microTESE technique, with its seminiferous tube visualization step, may certainly reduce this variability and make the biopsy results more reproducible.

Despite these limitations, the RF model showed excellent performance and seemed to be the most appropriate modeling approach. Additionally, 120 patients seemed adequate as a study population to train and validate this model. A diagnostic tool with perfect performance would be difficult to obtain, so a model with an AUC of nearly than 90% and an excellent sensitivity would probably be suitable as an additional tool for the management of men presenting with azoospermia. It would, therefore, be necessary to confirm these results and improve the performance of the model in a prospective multicenter design study, for sufficient robustness.

### Conclusions

ML models can provide the basis for an enhanced decision support system tool in the context of azoospermia, as they give additional and more relevant information than each variable taken separately. Nevertheless, many innovative AI models were tested, but none can determine TESE results with absolute certainty. However, we report promising results with decision tree–based ensemble models, and these require multicentric validation prior to any clinical use. It would, therefore, be interesting to improve the model, and several ways for improvement should be considered. Indefinitely increasing the number of participants and testing other DL models do not seem to be the most effective solutions. Integrating additional innovative biomarkers into the models may be necessary to improve the model’s performance. For example, biomarkers in seminal plasma may be of interest, as well as small noncoding RNA, which are increasingly being studied and whose variation in seminal plasma could encourage new diagnostic perspectives.

## Appendix

Multimedia Appendix 1 Most performing hyperparameter combination for each model.

Multimedia Appendix 2 The importance of each variable in the LR, BNC, RF, GBT, XGB, SVM, and KNN models model using permutation feature importance (PFE).

Multimedia Appendix 3 Learning curve for the logistic regression (LR), Bayesian naive classification (BNC), gradient-boosted tree (GBT), XGBoost (XGB), support vector machine (SVM), and k-nearest neighbor (KNN) models: graphical representation of the relationship between model performance (measured on the vertical axis (mean area under the curve [AUC]) and the number of patients used for training (on the horizontal axis).

## Abbreviations

ANN: artificial neural network
AUC: area under the curve
AZF: azoospermia factor
cTESE: conventional surgical testicular sperm extraction
DL: deep learning
FSH: follicle-stimulating hormone
GBT: gradient-boosted tree
ICSI: intracytoplasmic sperm injection
LH: luteinizing hormone
LR: logistic regression
microTESE: microsurgical testicular sperm extraction
ML: machine learning
NOA: nonobstructive azoospermia
PFE: permutation feature importance
RF: random forest
ROC: receiver operating characteristic
SVM: support vector machine
TESE: testicular sperm extraction
TRIPOD: Transparent reporting of a multivariable prediction model for individual prognosis or diagnosis

## Data Availability

The data sets generated during and/or analyzed during the current study are available from the corresponding author upon reasonable request.
