# Supplementary material for: A Machine Learning Approach for the Prediction of Testicular Sperm Extraction in Nonobstructive Azoospermia: Algorithm Development and Validation Study
Source: J Med Internet Res. 2023 Jun 21;25:e44047. doi: 10.2196/44047 (PMC10337455; doi:10.2196/44047)
Supplement: Multimedia Appendix 1 [file jmir_v25i1e44047_app1.docx]

**Table S1.** Most performing hyperparameters combination for each model. Hyperparameters not mentioned are set by default.

Bayesian naive classification [BNC], logistic regression [LR], k-nearest neighbor classifier [KNN], support vector machine [SVM], random forest [RF], gradient boosting trees [GBT], and Extreme Gradient Boosting [XGB].

| **Model** | **Param #1** | **Param #2** | **Param #3** | **Param #4** | **Param #5** | **Param #6** |
| --- | --- | --- | --- | --- | --- | --- |
| LR | penalty = l2 | c = 0.1 | solver = lbfgs | max_iter = 1000 |  |  |
| BNC | var_smoothing = 1e-8 |  |  |  |  |  |
| RF | bootstrap = true | max_depth = 70 | max_features = auto | min_samples_leaf = 1 | min_samples_split = 2 | n_estimators = 2000 |
| GBT | n_estimators = 500 | max_depth = 9 | learning_rate = 0.1 |  |  |  |
| XGB | max_depth = 4 | min_child_weight = 5 | learning_rate = 0.1 | n_estimators = 100 |  |  |
| SVM | c = 0.1 | kernel = poly | gamma = 0.1 |  |  |  |
| KNN | n_neighbors = 5 | weights = uniform | metric = minkowski |  |  |  |
|  |  |  |  |  |  |  |
