# Supplementary material for: A Machine Learning Approach for the Prediction of Testicular Sperm Extraction in Nonobstructive Azoospermia: Algorithm Development and Validation Study
Source: J Med Internet Res. 2023 Jun 21;25:e44047. doi: 10.2196/44047 (PMC10337455; doi:10.2196/44047)
Supplement: Multimedia Appendix 2 [file jmir_v25i1e44047_app2.docx]

**Table S2.** The importance of each variable in the LR, BNC, RF, GBT, XGB, SVM, and KNN models model using permutation feature importance (PFE); Data presented as mean (standard deviation). Variables located at higher ranks have more impact on the model predictions.

Bayesian naive classification [BNC], logistic regression [LR], k-nearest neighbor classifier [KNN], support vector machine [SVM], random forest [RF], gradient boosting trees [GBT], and Extreme Gradient Boosting [XGB].

| **Model** | **Age** | **BMI** | **smoking** | **FSH** | **LH** | **Testosterone** | **Inhibin B** | **Prolactin** |
| --- | --- | --- | --- | --- | --- | --- | --- | --- |
| LR | 3.03 (1.02) | 1.49 (1.12) | 3.31 (1.27) | 9.77 (2.22) | 3.60 (1.17) | 0.97 (1.02) | 9.31 (2.14) | 0.00 (0.00) |
| BNC | 2.00 (0.86) | 0.80 (0.78) | 0.00 (0.00) | 2.29 (1.68) | 1.54 (1.81) | 0.00 (0.00) | 3.49 (1.29) | 1.49 (0.82) |
| RF | 3.20 (0.78) | 1.94 (0.82) | 0.00 (0.00) | 1.03 (0.80) | 0.51 (0.47) | 0.86 (0.46) | 26.57 (2.47) | 3.09 (1.09) |
| GBT | 2.51 (0.64) | 2.69 (0.85) | 0.00 (0.00) | 2.80 (1.16) | 1.14 (0.68) | 1.43 (0.46) | 32.40 (2.69) | 5.94 (1.31) |
| XGB | 4.06 (1.34) | 1.43 (0.38) | 0.00 (0.00) | 1.83 (0.71) | 1.31 (0.77) | 2.97 (1.25) | 32.63 (2.59) | 5.94 (1.77) |
| SVM | 1.26 (0.91) | 1.43 (1.12) | 3.09 (1.18) | 5.14 (1.84) | 0.29 (1.06) | 2.00 (1.06) | 5.20 (1.52) | 2.17 (1.25) |
| KNN | 1.94 (1.31) | 0.40 (0.99) | 0.00 (0.00) | 3.89 (2.14) | 1.49 (1.95) | 0.00 (0.00) | 2.34 (2.18) | 0.80 (1.56) |
|  |  |  |  |  |  |  |  |  |

| **Model** | **Normal karyotype** | **Y microdeletion** | **Cryptorchidism** | **Infection** | **Trauma** | **Gonadotoxic therapy** | **Urogenital Surgery** | **Varicocele** |
| --- | --- | --- | --- | --- | --- | --- | --- | --- |
| LR | 1.94 (1.23) | 0.86 (0.46) | 2.74 (0.67) | 2.51 (1.52) | 0.00 (0.00) | 1.71 (0.81) | 0.00 (0.00) | 4.86 (1.18) |
| BNC | 0.97 (0.85) | 0.00 (0.00) | 0.29 (0.64) | 1.66 (1.26) | 0.17 (0.51) | 0.74 (0.73) | 0.34 (0.86) | 2.80 (1.39) |
| RF | 0.00 (0.00) | 0.51 (0.17) | 0.57 (0.00) | 0.00 (0.00) | 0.00 (0.00) | 0.00 (0.00) | 0.00 (0.00) | 2.34 (0.40) |
| GBT | 0.00 (0.00) | 0.69 (0.43) | 2.91 (0.31) | 0.00 (0.00) | 0.00 (0.00) | 0.00 (0.00) | 0.00 (0.00) | 3.09 (0.73) |
| XGB | 0.00 (0.00) | 0.00 (0.00) | 2.11 (0.45) | 0.00 (0.00) | 0.00 (0.00) | 0.00 (0.00) | 0.00 (0.00) | 1.26 (0.34) |
| SVM | 4.34 (0.64) | 3.43 (0.72) | 2.51 (0.58) | 1.37 (1.00) | 1.03 (0.43) | 0.63 (0.47) | 0.74 (0.37) | 3.94 (1.43) |
| KNN | 3.77 (1.03) | 2.69 (0.63) | 0.00 (0.00) | 0.29 (0.78) | 0.17 (0.51) | 0.86 (0.93) | 0.00 (0.00) | 4.69 (1.40) |
