# Supplementary figures and images for: A Machine Learning Approach for the Prediction of Testicular Sperm Extraction in Nonobstructive Azoospermia: Algorithm Development and Validation Study
Source: J Med Internet Res. 2023 Jun 21;25:e44047. doi: 10.2196/44047 (PMC10337455; doi:10.2196/44047)

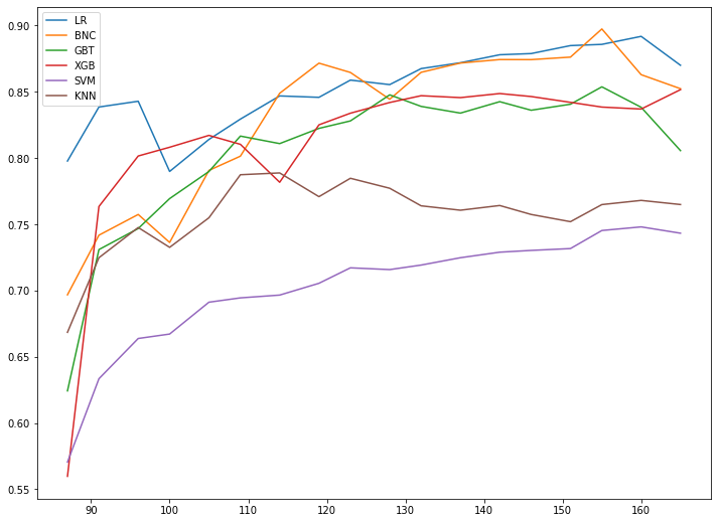

Supplement: Multimedia Appendix 3 [file jmir_v25i1e44047_app3.png]
